# Notes and News

**Published:** 1902-06-07

**Authors:** 


### Notes and News.

The King presented the medals of the Association to 35 representative members of the St. John's Ambulance Association who have served during the war in South Africa, on Saturday, at Buckingham Palace. The Association sent out 2,000 men. A tablet to the memory of those of the Metropolitan Corps who died in South Africa has been erected in St. Paul's Cathedral.

The International Congress for the Protection of Children will be held in London this year, during July, and will be under the patronage of the King. It will be divided into three sections, medical, legislative, and educational.

The Duke of Portland has formally opened the new Consumption Sanatorium for Nottingham near Mansfield, in the district of Sherwood Forest. The sanatorium, which is for 24 inmates, has cost £5,000. It has been occupied since the beginning of the year.

The Usher Institute of Public Health will be presented, on June 11th, to the university of Edinburgh, by Sir John Usher, who presented £8,000 also towards the endowment of the New Chair of Public Health and Sanitary Science at the University of Edinburgh.

Canada is waking up to the disadvantage of receiving diseased emigrants amongst its population. A Bill is now before the House of Commons to amend the Immigration Act. It aims at prohibiting the landing of persons suffering from dangerous and infectious diseases, except temporarily for treatment. The subject of consumption is receiving special attention in Canada, and two gentlemen in Montreal and Ottawa have offered to construct sanatoria at their own expense.

According to the annual report of the Registrar-General which has just been issued, the marriage rate for the year 1900 was 16·0 married per 1,000, the mean rate for 1890-99 having been 15·6. The lowest marriage rate was 11·9 in Rutland, and the highest 18·0 in London. The birth rate was 28·7 per 1,000, and was the lowest rate on record, being 1·3 per 1,000 below the mean rate for 1890-99. The lowest birth rate was 21·9 in Rutland, and the highest 35·3 in Durham. The lowest proportion of infants born out of wedlock was 27 in Essex, and the highest 67 in Westmorland.

The *Medical News* of New York says that Congress will pay the funeral expenses of President McKinley, including the physicians' bills over which there has been so much contention. An item is to be inserted in a Bill now under consideration which provides for an appropriation of $50,000 to defray the expenses attending the death and burial of the President, and it is understood that an agreement has been reached whereby $31,000 of this amount shall go to the physicians and the remainder shall be used to defray the funeral expenses. Friends of the dead President and others interested have been consulted, and it is believed that the allowance will be entirely satisfactory to all concerned.

During the debate on the Estimates a question came up in regard to the refusal by certain magistrates of certificates under the conscience clause of the Vaccination Acts, in answer to which Mr. Ritchie stated that it was impossible for any Home Secretary to tell magistrates that they must be satisfied of the conscientious nature of an applicant's objection to vaccination when as a fact they were not satisfied. The law said they must be satisfied on this point. We may add that, although in certain districts they may have been difficult to convince, in many places, including the metropolis, magistrates have displayed quite a touching confidence in the veracity of the applicants and in the depth of their convictions on the subject.

A good deal of excitement has been caused by the erection of a small-pox hospital in the neighbourhood of Hanworth, in Middlesex, and by the statement that the sewage of this hospital must flow into the River Thames in the neighbourhood of the intakes of some of the water companies. In answer to a question in Parliament Mr. Long has stated that there is no fear of the sewage entering the river "provided that the cesspools on the site are emptied regularly," and further that "there is no scientific evidence that small-pox infection can be conveyed by water." This assurance does not seem to have given much comfort to the objectors, who, however, while protesting so strongly about what is under their noses, seem to forget that in drinking Thames water at all they are of necessity drinking the sewage of a vast district which is highly farmed, densely inhabited, and thickly planted with towns and villages. If the arrangements for the filtration of the water cannot manage the one infection, how about all the rest? We may reasonably believe that the suggestion about the water has merely been put forward as a bogie by the people in the district with the object of inducing Londoners to protest against the site of the hospital.

